# Food insecurity and unmet healthcare needs in South Korea

**DOI:** 10.1186/s12939-023-01937-z

**Published:** 2023-08-04

**Authors:** Hwi Choe, Tae-Young Pak

**Affiliations:** Department of Consumer Science and Convergence Program for Social Innovation, Sungkyunkwan University, Seoul, South Korea

**Keywords:** Food insecurity, Food insufficiency, Hunger, Healthcare access, Healthcare equity, Forgone medical care, South Korea

## Abstract

**Background:**

Food insecurity is a significant risk factor for chronic and infectious diseases. It is also a barrier to accessing healthcare because food insecurity tends to co-occur with other socioeconomic disadvantages. The objective of this study is to examine whether food insecure individuals in South Korea can access desired level of healthcare when needed.

**Methods:**

This repeated cross-sectional study used data from the 2013–2015 and 2019–2021 waves of the Korean National Health and Nutrition Examination Survey. Multivariable logistic regression models were used to examine the association between household food insecurity and two indicators of unmet healthcare needs - any experience of forgoing medical service and the reasons for unmet needs (problems with availability, acceptability, and accessibility). Covariates indicating predisposing, enabling, and need factors were included in the regression analyses.

**Results:**

Of the 19,394 participants aged 19–64 years, 4.5% were moderately food insecure, 0.9% were severely food insecure, and 9.3% reported unmet healthcare needs. In the adjusted model, moderate food insecurity (OR, 1.47; 95% CI, 1.19–1.82) and severe food insecurity (OR, 3.32; 95% CI, 2.27–4.85) were associated with higher odds of unmet healthcare needs in a dose-graded manner. These associations were largely due to the increased odds of accessibility-related unmet needs among participants with moderate (OR, 2.31; 95% CI, 1.68–3.19) and severe food insecurity (OR, 6.15; 95% CI, 3.91–9.68).

**Conclusions:**

Food insecurity was associated with higher odds of unmet healthcare needs among Korean adults. Competing life demands may have a cumulative impact on health over the short and long term. Efforts to address trade-offs between healthcare needs and food insecurity may improve the health and well-being of marginalized populations.

## Background

Ensuring universal access to healthcare is an internationally recognized goal, proposed by the United Nations’ agenda for sustainable development [1]. However, inequalities in healthcare access persist across marginalized populations, with poorer individuals having the least entitlement to medical services [2]. “Unmet healthcare need” is a multifaceted construct that refers to the gap between healthcare needs and the services actually received [3]. It is defined as “a failure to obtain care when people believed it to be medically necessary” [4], thereby increasing morbidity and mortality risks [5]. Reasons for unmet healthcare needs include problems with availability (e.g., long waiting time, shortage of services), accessibility (e.g., financial and transportation barriers), and acceptability (e.g., attitudes toward care, competing responsibilities) of services. In 2018, approximately 7.8–10.8% of the Korean population reported that they could not obtain the medical services that they needed, and 1.2–2.5% reported having unmet healthcare needs due to cost [6].

Food insecurity, defined by the U.S. Department of Agriculture as “limited or uncertain availability of nutritionally adequate and safe foods, or limited or uncertain ability to acquire acceptable foods in socially acceptable ways” [7], is an important social determinant of health. Difficulty in access to food harms health by promoting the intake of cheap and processed foods [8] and forces people to forgo required medical services [9]. Empirical research has demonstrated a link between food insecurity and adverse health conditions, including cardiovascular disease [10–12], diabetes [8, 13, 14], allostatic load [15, 16], mental illness [17–20], and reduced grip strength [19]. Prior research has identified 5.4% of Korean adults as food insecure, with 1.1% having experienced disrupted eating patterns or reduced food intake [21]. Food insecurity is closely related to other socioeconomic disadvantages, which compound the healthcare burden on low-income households [22].

Coping with food insecurity poses a unique challenge to low-income households as they face a greater need for medical services but have limited financial resources and insurance coverage. Individuals in food insecure households are more likely to use medical services while spending larger amounts on healthcare [23]. However, they often postpone necessary care because of competing demands on their limited financial resources. Food insecurity tends to co-occur with other socioeconomic challenges, such as housing instability [23] and job insecurity [24], which could further tighten healthcare budgets [25]. Prior research has documented an association between food insecurity and healthcare underuse in at-risk populations in the US [9, 23, 26].

In the current study, we aim to understand the extent of unmet healthcare needs among food-insecure households in Korea. Despite Korea’s universal healthcare system, the citizens continue to pay a large share of medical expenses out of pocket. In 2019, the share of healthcare in total household consumption was more than 5%, which is higher than the OECD average of 3.1% [27]. The co-payment burden is disproportionately higher for low-income households as they face a greater need for costly medical treatments [28]. The dual challenges of healthcare financing and food insecurity are critically important, but they were yet to be fully understood in the Korean context. To fill this gap in the literature, we conduct a retrospective, population-based cohort study investigating the association between food insecurity and unmet healthcare needs among Korean adults. We hypothesize that (a) food insecurity is associated with unmet healthcare needs adjusted for sociodemographic covariates and that (b) the association is dose graded so that severe food insecurity is associated with greater unmet needs for medical services. We also examine the reasons for unmet needs to examine the mechanism through which food insecurity restricts healthcare access.

## Methods

### Data description

Data were drawn from the 2013–2015 and 2019–2021 waves of the Korean National Health and Nutrition Examination Survey (KNHANES).^1^ The KNHANES is an annual cross-sectional survey administered by the Korea Centers for Disease Control and Prevention (https://knhanes.kdca.go.kr/knhanes/main.do). The survey employed a multistage stratified cluster sampling method to obtain a nationally representative sample of the Korean population. The questionnaire consists of three modules: a health interview, physical examination, and nutrition survey. These surveys offer data on sociodemographic factors, health and health behaviors, healthcare access, and nutritional characteristics that include food insecurity. The study sample was restricted to participants aged 19–64 years who provided valid responses to the variables used for the analyses. Those older than 64 years were removed from the sample to minimize the confounding effects of retirement [29] and old age pension [30] on health and healthcare. The final sample includes 19,394 participants.

### Unmet healthcare needs

The outcome variables include (a) an indicator of self-perceived unmet need for healthcare and (b) indicators of the reasons for unmet healthcare needs. Any unmet healthcare need was assessed with a question, “*During the past 12 months, have you felt you needed healthcare but did not receive it?*”. Participants who answered yes to this question were determined to have unmet healthcare needs in the past year. Those who answered no were considered to have fulfilled their treatment needs.

Participants who provided an affirmative response were further asked about the reasons for their unmet healthcare needs. Seven possible reasons were presented along with an open-ended response option to indicate one’s own reason (Table 1). These response options are classified into three distinct categories: problems with availability, acceptability, and accessibility of medical services [31]. Unmet healthcare needs due to limited availability indicate a situation in which healthcare access is restricted owing to supply side problems (e.g., long waiting time and difficulty in making reservations). Unmet healthcare needs due to limited acceptability are related to personal preferences or circumstances (e.g., not having enough time, symptoms not severe, and afraid of medical tests or treatments). Finally, unmet healthcare needs due to limited accessibility include cost-related concerns and restrictions on transportation to get to healthcare providers. We defined binary indicators for each subcategory to confirm the source of unmet healthcare needs in response to food insecurity. Unmet healthcare needs due to availability and accessibility issues have strong policy implications as they can be modified by the government and health authorities.

**Table 1:** Classification of unmet healthcare needs

Category of unmet need	Stated reasons for unmet need	N (%)
Any unmet need		1894(9.3)
Availability unmet	Waiting time too long	107(0.5)
	Difficult to make reservations	
	Others^a^	
Acceptability unmet	Symptoms not severe	1370(6.9)
	Did not have time	
	Afraid of medical tests or treatments	
	Others^b^	
Accessibility unmet	Cost	417(1.9)
	Transportation	
	Others^c^	

*Notes*: N, number of participants. All frequency figures are unweighted; percentages are weighted

^a^Other reasons due to limited availability include inadequate medical services, distrust in the Korean healthcare system, no hospitals nearby, no specialty care offered in area

^b^Other reasons due to limited acceptability include concerns of coronavirus infection, pregnancy, breastfeeding, lack of energy to visit hospital, no evidence of recovery, too busy, didn’t know where to go, decided not to seek care, etc

^c^Other reasons due to limited accessibility include no health insurance coverage and impaired mobility

### Food insecurity

Food insecurity was measured using an 18-item questionnaire scale obtained from the U.S. Household Food Security Survey Module. The scale consists of three items on household food conditions, seven items on food access of households with adults only, and eight items on food access of households with children (Appendix, Table A1). These items are designed to measure food insecurity caused by resource constraints in the preceding year. Affirmative responses indicating inadequate food access were assigned a score of 1, and all other responses were assigned a score of 0. Binary scores were summed to construct an aggregate score for household food insecurity. The aggregate score ranges from 0 to 10 for households without children and from 0 to 18 for households with children.

Households with an aggregate score of 0–2 were determined to be food secure. An aggregate score of 3 to 5 for households without children or a score of 3 to 7 for households with children under the age of 18 years was categorized as having moderate food insecurity. A score of 6 to 10 for households with no children or a score of 8 to 18 for households with children was defined as having severe food insecurity. The Cronbach’s α for the food insecurity scale was 0.85 in the study sample. A detailed description of the scale development and validation is available in the literature [32, 33].

### Statistical analysis

Multivariable logistic regression analysis was estimated to examine the association between unmet healthcare needs and household food insecurity. Given that this study examines two types of outcome variables, i.e., any unmet healthcare need and the reasons for unmet healthcare needs, we estimated two sets of logistic regressions corresponding to each outcome variable. In all regressions, the food secure group was omitted as a reference category, and moderate and severe food insecurity were included along with covariates. Coefficient estimates were shown as odds ratios (OR) with a 95% confidence interval (CI). All analyses were adjusted using the sampling weights for the nutrition submodules in the KNHANES. Statistical significance was determined as *p* < 0.05. All analyses and estimations were performed using STATA SE version 17.0 (Stata Corp, College Station, TX).

### Covariates

Demographic and socioeconomic covariates were selected according to Andersen’s behavioral model of healthcare utilization [34]. Andersen’s model postulates that predisposing, enabling, and need factors jointly determine the medical service use of vulnerable populations. Predisposing factors represent individuals’ baseline characteristics that may determine health risks and healthcare service needs [35]; examples include age, gender, marital status, educational attainment, and employment status. Enabling factors include resource-related impediments or enablers [35], such as household income, home ownership, household size, health insurance ownership, urbanicity, housing type, and city/province of residence. Need factors determine one’s need and propensity to access healthcare [35] and include doctor-diagnosed chronic conditions (arthritis, asthma, diabetes, hypertension)^2^ and self-rated health.

Age was calculated as the year of the survey minus the year of birth. Education attainment was recorded as ≤ middle school, high school, and ≥ college education. Marital status was categorized as married, single, separated, divorced, and widowed. Employment status was divided into four mutually exclusive categories: full-time workers, temporary or daily workers, employers or self-employed, and unemployed or retired. Household income is the monthly average income of all household members reported in 10k KRW. Chronic conditions were binary coded for each disease diagnosed by a doctor. Self-rated health was categorized as poor and good or better.

## Results

The average sample statistics for 11,500 women [50.4%] and 7894 men [49.6%] are presented in Table 2. All frequency figures are unweighted; percentages, means, and standard errors are weighted. The mean [SD] age of the participants was 41.8 [12.6] years. A total of 13,717 [66.0%] participants were married, and 8626 [47.2%] were college-educated. There were 7030 [38.8%] full-time wage workers, 2449 [12.6%] temporary workers, and 6934 [33.3%] unemployed or retired. By food security status, 191 [0.9%] were classified as having severe food insecurity, 925 [4.5%] with moderate food insecurity, and 18,278 [94.6%] with food security. The prevalence of food insecurity was higher in the group that reported unmet healthcare needs in the preceding 12 months (249 [11.3%]) than in the other groups with sufficient healthcare access (867 [4.9%]).

**Table 2:** Descriptive statistics of study sample

		Number of participants (%)			
Variables	No unmet need (N = 17,500)	Any unmet need (N = 1894)	*P*-value	Total (N = 19,394)	
**Food insecurity status**			< 0.01		
Severe	117(0.7)	74(3.2)		191(0.9)	
Moderate	750(4.2)	175(8.1)		925(4.5)	
Secure	16,633(95.2)	1645(88.7)		18,278(94.6)	
**Predisposing factors**					
Age: mean (SD)	41.9(12.6)	41.5(12.6)	0.24	41.8(12.6)	
Gender			< 0.01		
Female	10,195(49.4)	1305(59.6)		11,500(50.4)	
Male	7305(50.6)	589(40.4)		7894(49.6)	
Marital status			< 0.01		
Married	12,448(66.3)	1269(63.4)		13,717(66.0)	
Single	3748(27.8)	401(27.9)		4149(27.8)	
Separated/widowed/divorced	1304(5.9)	224(8.7)		1528(6.1)	
Education attainment			< 0.01		
College or higher	7873(47.6)	753(43.7)		8626(47.2)	
High school	6850(40.5)	696(39.0)		7546(40.4)	
Middle school or below	2777(11.9)	445(17.3)		3222(12.4)	
Employment status			< 0.01		
Full-time worker	6425(39.3)	605(34.8)		7030(38.8)	
Temporary or daily worker	2171(12.3)	278(15.3)		2449(12.6)	
Employer or self-employed	2681(15.1)	300(16.4)		2981(15.2)	
Unemployed or retired	6223(33.3)	711(33.5)		6934(33.3)	
**Enabling factors** ^a^					
Household income (monthly)	498.8(316.1)	425.8(291.8)	< 0.01	491.9(314.7)	
Home ownership			< 0.01		
Over two	2459(13.3)	220(11.6)		2679(13.2)	
One	9167(51.7)	890(45.5)		10,057(51.1)	
None	5874(35.0)	784(42.9)		6658(35.7)	
Household size			0.02		
One	1356(7.4)	173(8.2)		1529(7.5)	
Two	4122(20.2)	445(20.7)		4567(20.2)	
Three	5025(30.2)	575(31.6)		5600(30.3)	
Over four	6997(42.2)	701(39.6)		7698(41.9)	
Private health insurance			< 0.01		
Yes	15,700(89.7)	1624(86.6)		17,324(89.4)	
No	1800(10.3)	270(13.4)		2070(10.6)	
Urbanicity			0.36		
Urban	14,593(86.5)	1564(85.9)		16,157(86.4)	
Rural	2907(13.5)	330(14.1)		3237(13.6)	
Housing type			< 0.01		
Apartment	10,219(57.7)	982(52.1)		11,201(57.2)	
Non-apartment	7281(42.3)	912(47.9)		8193(42.8)	
**Need factors**					
Arthritis			< 0.01		
Yes	1160(4.9)	213(8.7)		1373(5.3)	
No	16,340(95.1)	1681(91.3)		18,021(94.7)	
Asthma			< 0.01		
Yes	433(2.6)	75(3.7)		508(2.7)	
No	17,067(97.4)	1819(96.3)		18,886(97.3)	
Diabetes			0.70		
Yes	997(5.0)	112(4.6)		1109(5.0)	
No	16,503(95.0)	1782(95.4)		18,285(95.0)	
Hypertension			0.06		
Yes	2443(11.8)	235(10.1)		2678(11.7)	
No	15,057(88.2)	1659(89.9)		16,716(88.3)	
Self-rated health			< 0.01		
Good or better	15,067(86.8)	1281(69.3)		16,348(85.2)	
Poor	2433(13.2)	613(30.7)		3046(14.8)	

*Notes*: N, number of participants; SD, standard deviation. All frequency figures are unweighted; percentages, means, and standard errors are weighted

^a^City/province of residence is omitted for brevity

Table 3 reports results from the logistic regression of any unmet healthcare need on food insecurity, adjusted for predisposing, enabling, and need factors. The model based on the full sample shows that being moderately food insecure is associated with 1.47 (95% CI, 1.19–1.82) times higher odds of any unmet healthcare need, and that being severely food insecure is associated with 3.32 (95% CI, 2.27–4.85) times higher odds of any unmet healthcare need. For women, moderate food insecurity is associated with odds ratio for any unmet healthcare need of 1.72 (95% CI, 1.35–2.20), and severe food insecurity is associated with odds ratio of 3.36 (95% CI, 2.07–5.44). For men, moderate food insecurity is associated with 1.10 (95% CI, 0.73–1.66) times higher odds of any unmet healthcare need, and severe food insecurity is associated with 3.38 (95% CI, 1.86–6.15) times higher odds of any unmet need.

**Table 3:** Associations between food insecurity and any unmet healthcare need, multivariate logistic regression results

Sample:	Total		Women		Men	
	OR (95% CI)	*P*-val.	OR (95% CI)	*P*-val.	OR (95% CI)	*P*-val.
Severe food	3.32		3.36		3.38	
insecurity	(2.27, 4.85)	< 0.01	(2.07, 5.44)	< 0.01	(1.86, 6.15)	< 0.01
Moderate food	1.47		1.72		1.10	
insecurity	(1.19, 1.82)	< 0.01	(1.35, 2.20)	< 0.01	(0.73, 1.66)	0.65
Food security	Referent		Referent		Referent	

*Notes*: OR, odds ratio; CI, confidence interval. Regressions control for age, gender, marital status, education attainment, employment status, household size, household income, home ownership, private health insurance, urbanicity, housing type, chronic conditions (arthritis, asthma, diabetes, hypertension), self-rated health, and year of survey

The following results show the logistic regression of the three subcategories of unmet needs (availability, acceptability, and accessibility unmet) on food insecurity, adjusted for predisposing, enabling, and need factors (Table 4). Moderate (OR, 1.21; 95% CI, 0.49–2.98) and severe (OR, 0.70; 95% CI, 0.09–5.61) food insecurity are not associated with having availability-related unmet needs. Similarly, moderate (OR, 1.01; 95% CI, 0.76–1.34) and severe (OR, 1.06; 95% CI, 0.58–1.92) food insecurity are not associated with having acceptability-related unmet needs. However, moderate food insecurity is associated with 2.31 (95% CI, 1.68–3.19) times higher odds of accessibility-related unmet needs, and severe food insecurity is associated with 6.15 (95% CI, 3.91–9.68) times higher odds of accessibility-related unmet needs.

**Table 4:** Associations between food insecurity and the reasons for unmet healthcare need, multivariate logistic regression results

Outcome:	Availability unmet		Acceptability unmet		Accessibility unmet	
	OR (95% CI)	*P*-val.	OR (95% CI)	*P*-val.	OR (95% CI)	*P*-val.
Severe food	0.70	0.73	1.06	0.85	6.15	< 0.01
insecurity	(0.09, 5.61)		(0.58, 1.92)		(3.91, 9.68)	
Moderate food	1.21	0.67	1.01	0.96	2.31	< 0.01
insecurity	(0.49, 2.98)		(0.76, 1.34)		(1.68, 3.19)	
Food security	Referent		Referent		Referent	

*Notes*: OR, odds ratio; CI, confidence interval. Regressions control for age, gender, marital status, education attainment, employment status, household size, household income, home ownership, private health insurance, urbanicity, housing type, chronic conditions (arthritis, asthma, diabetes, hypertension), self-rated health, and year of survey

## Discussion

Using a nationally representative Korean sample, we demonstrate that the problem of food insecurity and unmet healthcare needs remains significant in Korea, with 5.4% reporting food insecurity and 9.3% reporting unmet healthcare needs in the past 12 months. The prevalence of food insecurity and unmet healthcare needs highlights the potential tradeoffs between securing healthy and nutritious meals and paying off medical bills among at-risk populations [9, 23, 26]. Previous studies have shown an association between economic insecurity and cost-related medical service underuse in the U.S. and European contexts [23, 36, 37]; however, to our knowledge, little is known about inadequate healthcare access among food-insecure households in Korea. With the growing interest in addressing patients’ socioeconomic concerns as a routine part of clinical practice, this study investigated the associations between food insecurity and unmet healthcare needs.

Our descriptive analyses found that 9.3% of the sample had experienced any unmet healthcare needs in the past year. Of the sample with unmet needs, 0.5% were due to limited availability of healthcare, 6.9% were due to limited acceptability, and 1.9% were due to cost or transportation-related concerns. The experience of any unmet need was more pronounced among women, unmarried individuals, less educated, and temporary workers. It was also associated with socioeconomic variables such as income, homeownership, housing type, and health insurance coverage. These results were similar to those of previous research, highlighting that the prevalence of unmet care needs systematically varies based on health and socioeconomic characteristics [38–40].

We also found that unmet care needs were more prevalent in respondents with arthritis and asthma. However, it showed no association with other chronic conditions such as diabetes and hypertension. The health belief model suggests that individuals will take action to control ill-health if they perceive they are susceptible to a condition, the condition would have serious effects, and taking action would be beneficial [41]. Arthritis and asthma may be perceived as less severe compared to diabetes and hypertension. Therefore, participants who face lower risk from less severe diseases like arthritis and asthma would exhibit reduced use of needed medical services.

Consistent with our research questions, food insecurity was associated with having experienced unmet healthcare needs in the past 12 months. This association arose in a graded manner, suggesting that severely food-insecure individuals were more likely to experience unmet healthcare needs. Splitting the sample by gender, we found that both moderate and severe food insecurity were associated with unmet healthcare needs among women. However, for men, only severe food insecurity was indicative of unmet healthcare needs. Examining the reasons for unmet needs showed that these associations were largely due to cost- and transportation-related concerns regarding healthcare access. Other reasons for unmet healthcare needs (limited availability and acceptability of medical services) were not associated with food insecurity.

This study contributes to prior evidence reporting an increased health risk among food insecure populations. Previous studies have documented the association between food insecurity and adverse health conditions, such as cardiovascular disease [10–12], diabetes [8, 13, 14], allostatic load [15, 16], mental illness [17–20], and reduced grip strength [19]. Additionally, researchers have explored the mortality implications of these health conditions at a population level [42, 43]. Interpreting our findings in relation to prior research suggests that unmet healthcare is the likely pathway underlying the links between food insecurity, chronic disease, and mortality. This channel is particularly salient in Korea, where the healthcare system is characterized by high co-payment for tertiary care and outpatient medical services [44]. In 2019, a typical Korean citizen paid 1065 USD or 30.3% of their annual healthcare costs out-of-pocket [45]. Co-payment rates were as low as 20% but could escalate to 50–60% for patients who frequently seek care at upper-level medical institutions [44]. Under the current healthcare system, unmet healthcare needs may occur more often in marginalized populations that have greater needs to treat life threatening conditions. As this group lacks access to costly treatment options, they might be exposed to greater risk of acute conditions and eventually mortality.

The policy response to food insecurity should aim to provide healthy and nutritious meals to at-risk populations while also incorporating fundamental measures that address systemic factors that create competing demands on household finances. Government nutrition assistance programs in Korea (congregate meal program and home-delivered meal services) mainly target older adults and those with disabilities. These two national programs and temporary food support from local authorities constitute the main pillars of food assistance in Korea [17, 46]. Currently, food-insecure adults in Korea have no standalone food aid packages that consistently provide adequate and nutritious meals over the long term [46]. Although income support programs provide supplemental cash benefits to resource-constrained households, they do not consider the competing needs for nutrition and healthcare faced by marginalized individuals and households. Thus, policy reforms need to consider concomitant healthcare challenges that arise with food insecurity and offer comprehensive interventions that address systemic factors contributing to food insecurity and healthcare underuse among low-income adults and their family members.

Our findings highlight the importance for healthcare providers to consider patients’ food insecurity and related socioeconomic challenges when developing treatment plans. Clinicians need to be aware that patients may forgo opportunities to receive appropriate care because of cost-related concerns and other competing needs and thus require more affordable medical services to complete the recommended treatment dose [9, 47]. Knowledge of patients’ socioeconomic backgrounds may allow referrals for nutritional support, food assistance benefits, and other government aids that enhance the clinical efficacy of treatments. Given that individual clinicians may not rectify the root causes of diseases, the government needs to implement an organized and holistic aid package that addresses socioeconomic risk factors for ill health.

This study has several limitations. First, food insecurity was measured at the household level, while healthcare access was assessed at the individual level. Given that the measurement of food insecurity involves a subjective assessment of food access, the way this variable is measured could vary depending on who in the household responded to the survey. If the respondent lacked knowledge about the family’s food access, the measurement could be inaccurate, potentially leading to attenuation bias. Second, the cross-sectional study design limits our ability to examine the changes in food access over time. Food insecurity exhibits a complex time pattern depending on the receipt and duration of food assistance benefits [48], and this fluctuation in food supply may lead to a comparable cyclical pattern in healthcare utilization among food assistance beneficiaries. As our data are not longitudinal, the time pattern in the association between food insecurity and healthcare access remains underexplored in this study. Third, although we controlled for a large number of covariates, other competing needs that could confound the association between food insecurity and healthcare access, such as housing instability, excessive medical expenses, and participation in social welfare program, were left out of the regressions. Controlling for additional confounders may help delineate the mechanisms that link food insecurity to unmet healthcare needs. Finally, our key measures were based on participants’ self-reports, which could be influenced by recall or social desirability bias [9, 26, 47]. Future research should consider using administrative data to obtain more direct measures of food insecurity and unmet healthcare needs.

### Conclusions

Food insecurity tends to co-occur with unmet healthcare needs among disadvantaged populations. In this nationally representative study of Korean adults aged 19–64 years, we found that food insecurity is independently associated with unmet healthcare needs in a prior year, and that severe food insecurity leads to greater unmet healthcare needs in a dose-graded manner. Among various reasons for unmet care needs, limited accessibility due to cost and transportation was related to food insecurity outcomes. Limited food access needs to be considered as a modifiable risk factor for unmet care needs and prioritized in government interventions. Policies that improve food access may have positive spillover effects on healthcare utilization and produce health benefits over the short and long term, especially for marginalized minority groups.

## Data Availability

All data available in the Korean National Health and Nutrition Survey website: https://knhanes.kdca.go.kr/knhanes.

## Appendix

**Table A1:** Measurement items for household food insecurity

HH1	In the last 12 months, how often have (you/your household) ran out of food because there was not enough money to buy foods? (often; sometimes; never; DK/refusal)
HH2	In the last 12 months, how often have (you/your household) worried about running out of food because there was not enough money to buy foods? (often; sometimes; never; DK/refusal)
HH3	In the last 12 months, how often have (you/your household) forgone balanced meals because there was not enough money to buy foods? (often; sometimes; never; DK/refusal)
AD1	In the last 12 months, have (you/other adults in your household) cut the size of meals or skip meals because there was not enough money to buy foods? (yes; no; DK/refusal)
AD2	If yes, how often did this happen? (almost every month; some months but not every month; only 1 or 2 months)
AD3	In the last 12 months, have you eaten less than you felt you should because there was not enough money to buy foods? (yes; no; DK/refusal)
AD4	In the last 12 months, have you ever hungry but couldn’t eat because there was not enough money to buy foods? (yes; no; DK/refusal)
AD5	In the last 12 months, have you lost weight because you could not eat enough due to a lack of money for foods? (yes; no; DK/refusal)
AD6	In the last 12 months, have (you/other adults in your household) ever not eat for a whole day because there was not enough money to buy foods? (yes; no; DK/refusal)
AD7	If yes, how often did this happen? (almost every month; some months but not every month; only 1 or 2 months)
CH1	In the last 12 months, how often have you fed your children with only a few kinds of low-cost food due to a lack of money to buy foods? (often; sometimes; never; DK/refusal)
CH2	In the last 12 months, how often have you not offered your children a balanced meal due to a lack of money to buy foods? (often; sometimes; never; DK/refusal)
CH3	In the last 12 months, how often have you not fed your children as needed because there was not enough money to buy foods? (often; sometimes; never; DK/refusal)
CH4	In the last 12 months, have you cut the size of your children’s meal due to a lack of money for foods? (yes; no; DK/refusal)
CH5	In the last 12 months, have your children skipped meals due to a lack of money for foods? (yes; no; DK/refusal)
CH6	If yes, how often did this happen? (almost every month; some months but not every month; only 1 or 2 months)
CH7	In the last 12 months, have your children been unable to eat even when hungry because there was not enough money to buy foods? (yes; no; DK/refusal)
CH8	In the last 12 months, have your children had to go without eating for a whole day because there was not enough money to buy foods? (yes; no; DK/refusal)

*Notes*: HH, household specific question; AD, adult specific question; CH, child specific question

## Footnotes

1 This study omits the 2016–2018 wave of the KNHANES, as it used different questions for food insecurity.

2 Other chronic health conditions (cancer, stroke, cardiovascular disease, osteoporosis, tuberculosis, and pulmonary disease) were not incorporated into empirical analyses due to the insufficient number of food insecure participants diagnosed with these conditions.
